# Quality assurance measurements for high‐dose‐rate brachytherapy without film

**DOI:** 10.1120/jacmp.v15i1.4586

**Published:** 2014-01-06

**Authors:** Paul A. Jursinic

**Affiliations:** ^1^ West Michigan Cancer Center Kalamazoo MI USA

**Keywords:** high‐dose‐rate brachytherapy, quality assurance

## Abstract

The purpose of this study was to develop new and modified tools that allow HDR brachytherapy quality assurance tests to be carried out efficiently without film, video cameras, stopwatches, and mechanical rulers; and to devise methods that use these new tools for daily and quarterly check procedures, which are efficient and provide increased accuracy compared to previous methods. The HDR brachytherapy system tested was the GammaMedplus iX, Ir‐192 HDR. Various catheters and treatment applicators designed for this system were tested. To measure the absolute position of the source, a simple tool was built that uses a Plexiglas frame, a template for applicator positioning, and a diode for radiation detection. For daily reproducibility and source strength tests, modifications were made of a Model 70008, HDR brachytherapy Ir‐192 quality assurance tool, which is used with the HDR‐1000‐Plus well‐type reentrant chamber. Measurement procedures and analysis protocols were developed that use the Microsoft Excel spreadsheet program. Independent determination of source positions was made with a computer video camera and radiochromic film. Using the new tool, for a straight catheter the measured source position is found to be within ±0.1mm of the mechanically set distance, for a ring applicator ±0.3mm, for a tandem ±0.2mm, and for an ovoid ±0.2mm. Using the modified insert, daily dwell position can be determined with an accuracy of 0.3 mm and timer accuracy can be determined with an accuracy of 0.3% over a 20 s time frame. The time needed to carry out quarterly tests is estimated to be reduced by two‐to four‐fold compared to previous methods. New and modified equipment and procedures have been developed for measuring HDR brachytherapy dwell position and dwell times efficiently with high accuracy. The equipment described in this work can be built and modifications can be made in most clinics. Location of dwell position can be determined in straight catheters and ring and ovoid applicators. Timer linearity and accuracy can be determined. Source strength can be confirmed. Measurement efficiency is improved compared to previous methods that used film, video cameras, mechanical rulers, and stopwatches.

PACS number: 87.56.Fc

## I. INTRODUCTION

One of the key responsibilities of a medical physicist is to implement a quality management program in the clinic. For a high‐dose‐rate (HDR) brachytherapy program one must assure that the delivered dose is accurate and reliable. This is contingent on knowing the following: activity of the radioactive source when the treatment is delivered, length of source connection tubes and catheters, accurate and reliable locations of the source dwell positions with respect to the patient anatomy, accurate and reliable residence time at each dwell position, and timer linearity. Recommended^1^, ^2^, ^3^, ^4^, ^5^, ^6^ methods include cylindrical ion chambers, well‐type reentrant chambers with HDR brachytherapy inserts,^7^, ^8^, ^9^ radiographic film, closed‐circuit TV(video) monitors with a position verification tool (Varian Medical Systems, Milpitas, CA), mechanical rulers, and stop watches.

One of the changes that has occurred in the past few years is that many clinics no longer have dark rooms and film processing equipment. As a result, the use of radiographic film for HDR brachytherapy quality assurance is no longer feasible. An alternative is to use radiochromic film.^(10)^ In this work, new and modified tools have been developed that allow HDR brachytherapy quality assurance tests to be carried out efficiently and accurately without film, video cameras, stopwatches, and rulers. Methods of use for daily and quarterly check procedures are presented.

## II. MATERIALS AND METHODS

### A. Apparatus

The HDR brachytherapy system that was used was the GammaMedplus iX Ir‐192 HDR (Varian Medical Systems, Milpitas, CA). The air kerma strength of the source was measured with a model HDR100Plus well chamber (Standard Imaging, Middleton, WI) that had been calibrated at the University of Wisconsin Dosimetry Calibration Laboratory an Accredited Dosimetry Calibration Laboratory.

**Figure 1:**
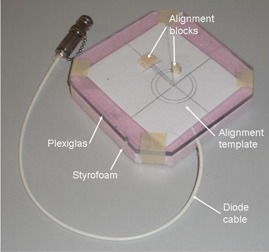
Apparatus that was constructed and used to measure the absolute position of the Ir‐192 source.

**Figure 2:**
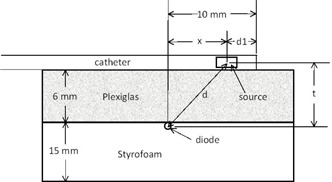
A diagram of the side view of the measurement apparatus. The distance of dwell position 1 from the outside surface of the catheter is d1. The distance from the position of the diode to the outside surface of the tip of the catheter is set at 10 mm by positioning the catheter against the stop bar shown in Fig. 1.

The absolute position of the source in a variety of catheters and a ring applicator was measured with the apparatus shown in Fig. 1 that was built in the clinic. The catheters and ring applicators were standard types (Varian Medical Systems) used with the GammaMedplus. Wooden blocks and an alignment template were glued on top of the apparatus to provide for easy, reproducible positioning of the catheters and ring applicator. The diode used in this apparatus was a P‐type semiconductor,^(11)^ designated as an SI4 (Standard Imaging). The diode was found to be sensitive to room light, so it was covered with black electrical tape. The diode output was measured with a Max4000 electrometer (Standard Imaging) operated in the zero‐bias mode. As shown in Fig. 2, the apparatus is a 6 mm thick piece of Plexiglas on top of a 15 mm thick piece of high‐density Styrofoam. A groove was cut into the Styrofoam to fit the diode and its cable. For the measurements in this work, the distance from the center of the catheter to the diode active area (t in Fig. 2) is 8.6 mm.

The GammaMedplus iX HDR brachytherapy system uses a connection tube that couples the HDR brachytherapy system to the catheter. The HDR brachytherapy system sends the source out 129.9 cm, which is the position of the first dwell position, $N_{\text{dwell}}=1$. The distance is measured from the position of an optical coupler inside the housing of the HDR brachytherapy system. The optical coupler is a light emitter and detector in a single assembly through which the source guide wire passes. When the end of the guide wire reaches optical coupler its optical path is broken and the brachytherapy system identifies the position of the end of the guide wire and measures the extension distance from that point. The connection tube between the HDR brachytherapy system housing and the catheter has an adjustable length with a threaded collar fitted with a lock nut. The combined length of the connection tube and any catheter is adjusted by comparison to a 130 mm long calibration wire. When the source is changed every three months, a measurement is made with the apparatus and the d1 value shown in Fig. 2, for $N_{\text{dwell}}=1$, is obtained. The position of the optic coupler in the HDR brachytherapy system is adjusted with a straight catheter in place so that d1 is 5.0 mm. This value of 5.0±0.2mm is based on the position of a first dwell position of a string of dummy seeds that are placed in a transparent straight catheter and measured with a ruler. The dummy seeds, model GM11009600 (Varian Medical Systems), are made of tungsten, are 1.1 mm long, have 10 mm center‐to‐center spacing, and are mounted on a plastic ribbon that is 360 mm long and has a 0.6 mm diameter.

The first dwell position can be offset with respect to the diode. This is accomplished by misadjusting the length of the source guide tube by turns of the threaded sleeve, whose pitch was measured with a micrometer and found to be 0.825 mm/turn.

The measurement template for straight and ring applicators is shown in Fig. 1. The center of the applicators is directly over the diode. For the straight catheter, the distance from the diode to the outside surface of the tip of the catheter is 10 mm. For the ring applicator, the distance of the path length from the diode along the center of the ring to the outside surface at the end of the ring is 10 mm.

**Figure 3:**
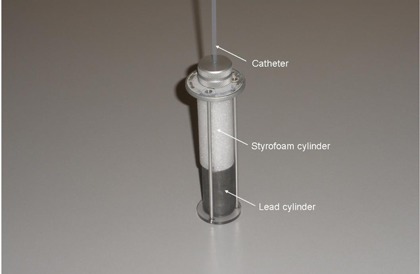
Modified HDR brachytherapy Ir‐192 quality assurance insert for the HDR 1000 Plus well chamber.

For recommended^4^, ^5^ daily source position reproducibility and source strength tests, the apparatus shown in Fig. 3 was used. This is a modified, Model 70008, HDR Ir‐192 quality assurance tool (Standard Imaging), used with the HDR‐1000‐Plus well‐type reentrant chamber (Standard Imaging). Use of this type of insert for quality assurance of HDR brachytherapy sources has been previously described.^(9)^ The HDR Ir‐192 quality assurance tool has two lead cylinders separated by a Plexiglas disk. For the modification shown in Fig. 3, the lead cylinder most proximal to the HDR brachytherapy system has been removed and replaced with a Styrofoam cylinder.

Photographs of the source position in a transparent catheter were made with a QuickCam Pro 4000, computer video camera (Logitech, Freemont, CA). The QuickCam Pro 4000 has a resolution of 1.3 Mpixels. GAFCHROMIC film, model EBT3, (Ashland Specialty Ingredients, Wayne, NJ) was used to image the source in applicators that are not transparent to visible light.

The calculation formalism and derivations of all equations are presented in the Appendix.

## III. RESULTS

**Figure 4:**
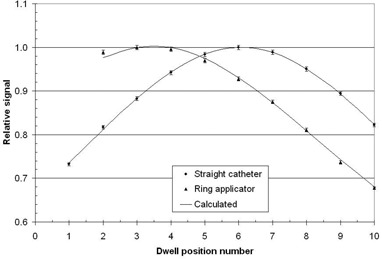
Measurements of a vaginal‐cylinder straight catheter and a 30° ring applicator with PLi of 1 mm. The signals are normalized to the maximum value. The points are measured values with error bars that indicate one standard deviation, which are based on five repeats of the experiment. The solid line is a fit to these measured data using Appendix Eqs. (1)‐(6). For the straight catheter d1=4.2±0.5mm and $\alpha =-0.001\pm 0.002\;{\text{mm}}^{-1}$, and for the ring applicator d1=8.2±0.3mm and $\alpha =-0.004\pm 0.002\;{\text{mm}}^{-1}$.

Measurements were made on a straight catheter for a vaginal‐cylinder applicator and are shown in Fig. 4. The modeling for the diode signal, Appendix Eqs. (1)‐(3), is very close to the measured data. For the straight catheter, the maximum difference between the measured and calculated signal is ±0.6% and the average difference is 0.0% with a standard deviation of 0.3%. For the ring applicator, the maximum difference between the measured and calculated signal is −1.4% and the average difference is −0.2% with a standard deviation of 0.7%.

For the straight catheter, a maximum in the signal occurs for dwell position 6, which will be directly over the diode, the closest position to the diode. The value of d1 is 5.2 mm for a straight catheter. It is also found that within experimental uncertainty a=0. This means that the attenuation of dose by the 6 mm thick Plexiglas is offset by the increase in scattered dose. This result is consistent with calculations^(12)^ of scatter dose being only 5% of total dose at 6 mm distance from an HDR brachytherapy Ir‐192 source.

**Figure 5:**
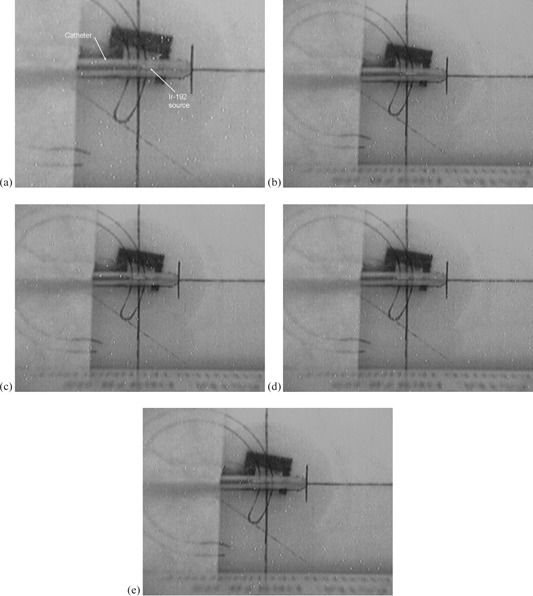
Photographs ((a) to (e)) of the Ir‐192 source in dwell positions 4 to 8, respectively. A straight catheter is in use and is placed in the apparatus with alignment according to the drawn template. The horizontal and vertical lines of the template indicate the position over the diode. The wooden stop blocks have been removed for clarity.

The straight catheter is transparent and this allows photographs to be taken for different positions of the Ir‐192 source. To independently verify the results of Fig. 4, closed circuit TV images were taken of the catheter for various dwell positions. Figures 5(a) to 5(e) show images for dwell positions 4 to 8, respectively. These photographs confirm the finding in Fig. 4 that dwell position 6, which is Fig. 5(c), is when the source is directly over the diode.

**Figure 6:**
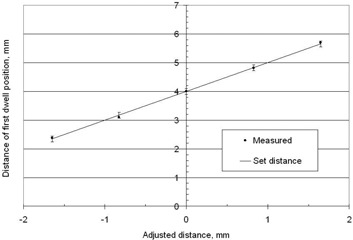
The distance of the middle of the first dwell position to the position over the diode for different mechanical settings of the length of the source guide tube with the threaded sleeve. The points are measured values with error bars that indicate one standard deviation, which are based on five repeats of the experiment.

Offsetting the first dwell position with respect to the diode can be used to test the sensitivity of the measurement technique. The results are shown in Fig. 6 with the measured dwell position being within ±0.1mm of the mechanically set distance.

Next, ring applicators were measured and typical data are shown in Fig. 4. For these measurements, the first dwell position is offset by 5 mm from the end travel position, as recommended by the Varian Company. Table 1 shows the results for ring applicators used in our clinic. A maximum in the signal occurs between dwell positions 3 and 4, which will be directly over the diode, the closest position to the diode. It is also found that within experimental uncertainty α=0.

**Figure 7:**
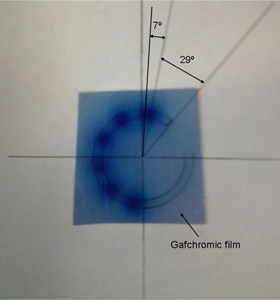
Radiograph of the Ir‐192 source in dwell positions 1 to 6, respectively. A 30° ring applicator is in use and is placed in the apparatus with alignment, according to the drawn template shown in Fig. 1, with a piece of GAFCHROMIC film under it.

To independently verify the results of Fig. 4, radiographic images were made with GAFCHROMIC film of the ring for various dwell positions. A radiograph is made of the 30° ring applicator and is shown in Fig. 7. The angular distance from the first dwell position to the diode is 7°, which is 1.9 mm and in good agreement with the data in Table 1 and Fig. 4.

Measurements were also made of ovoids and tandems used with ovoids and ring applicators. Table 2 shows the results of these measurements.

**Figure 8:**
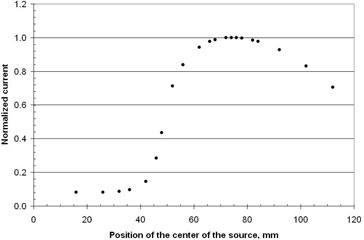
Relative current of the well ion chamber for different position of the source in the modified Model 70008 insert. The current is normalized to the maximum signal, 76.23 nA, which occurs at position 74 mm, dwell position 35. The position of the source is the distance from the middle of the source to the outer edge of the catheter.

For recommended^4^, ^5^ daily source position reproducibility and source strength tests, the catheter is placed in the modified Model 70008 insert, Fig. 3, which is placed in the well ion chamber. This is a modification of the insert described earlier.^(9)^ A procedure is run that has 21 different dwell positions, each with a 10 s dwell time. At each dwell position, the ion chamber current is measured. Typical signals are shown in Fig. 8. A low current occurs at positions less than 40 mm (dwell position number 21), which corresponds to the source being within the lead cylinder. The peak current, I(x=74), occurs at the distance from the middle of the source to the outer edge of the catheter, of 74 mm, which corresponds to dwell position 35. This dwell position is in the middle of the Styrofoam cylinder of the insert, Fig. 3. The current then decreases due to the source position moving away from the position of maximum sensitivity in the cavity of the well chamber. Dwell position 35 is an ideal location for measuring the source strength and its decay on a daily basis since it has only a small dependence on the position of the source.

**Figure 9:**
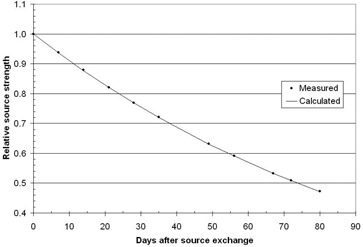
Measurement of the decay in source strength, using the method of Appendix Eq. (8). The points are measured values and the solid line is a calculation that uses the half time of 73.8 days.

Using the modified Model 70008 insert, the source strength of an Ir‐192 source was measured over a period of three months and the results are shown in Fig. 9.

**Figure 10:**
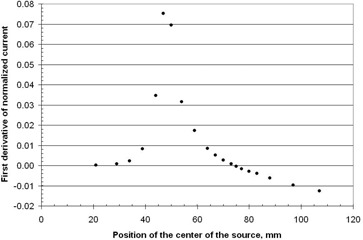
First derivative with respect to distance of the signal in Fig. 8. The position of the source is the distance from the middle of the source to the outer edge of the catheter.

To measure the position of the source, one needs a location with a high rate of change in current versus source position, which is described by Appendix Eq. (10). The first derivative of the data in Fig. 8 is shown in Fig. 10. The greatest value occurs at position 47 mm (dwell position number 21) in Fig. 10, which has a value of d(Sig(x=47,D)/dx=0.075signal/mm. The x’ position used is 74 mm, the position of maximum signal and, for the data in Figs. 8 and 10, Appendix Eq. (10) is evaluated as follows:

(1) $$\frac{\frac{d(I(x=47,D))}{\text{dx}}}{I(x\prime =74,D)}=0.075/76.19=9.84\times 10^{-4}{\text{mm}}^{-1}$$

**Table 1:** Measurements on ring applicators used in the clinic. The ring applicator angle is that of the straight shaft with respect to the plane of the ring. The indicated error ranges are one standard deviation, which is based on five repeats of the measurement

*Ring applicator*	*Distance offirst dwell position to the position over the diode (mm)*	*Distance offirst dwell to the outside edge of the end of the ring, dl (mm)*
30°	1.8±0.3	8.2±0.3
45°	0.9±0.3	9.1±0.3
60°	1.6±0.3	8.4±0.3

**Table 2:** Measurements of different applicators used in the clinic. The indicated error ranges are one standard deviation, which is based on five repeats of the measurement

*Applicator*	*Distance of first dwell to position over the diode (mm)*	*Distance of first dwell to the outside edge of the end of the tandem or ovoid, d1 (mm)*
Tandem used with ring	5.0±0.2	5.0±0.2
Tandem used with ovoid	6.0±0.2	4.0±0.2
Ovoid	6.0±0.2	4.0±0.2

The change in the absolute dwell position, Δx, compared to the day of source commissioning can be measured as follows by rearranging Appendix Eq. (10):

(2) Δx=(I(x=47,D)I(x=74,D)−I(x=47,D=0)I(x=74,D=0))9.84×10−4mm−1

**Figure 11:**
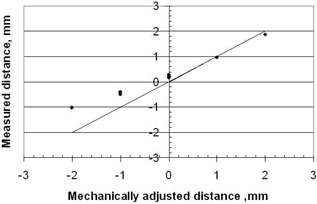
The measured distance of offset of dwell position 21 with respect to the edge of the lead insert shown in Fig. 3. The offset is varied with different mechanical settings of the source guide‐tube.

**Figure 12:**
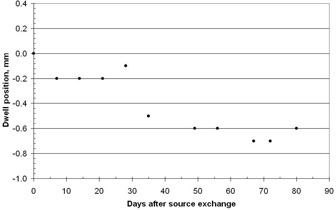
Measurement of the source dwell position over an 80 day period, using the method of Appendix Eq. (12).

The sensitivity of the position measurement can be tested by offsetting dwell position 21 at 47 mm with respect to the lead insert. This was accomplished by misadjusting the length of the source guide tube by turns of the threaded sleeve. The results are shown in Fig. 11. At mechanical distance zero the measured distance is 0.2 mm, which is the change in dwell position since the calibration at the last source change. Figure 12 shows the change in the dwell position measured between source changes. Also, the measured distance deviates from the mechanical distance as expected since the maximum first derivative is used and this is only true at position 47 mm in Fig. 10. Nevertheless, this is a sensitive method for detecting changes in absolute dwell position on a daily basis.

Recommended^4^, ^5^ daily checks of temporal accuracy can be accomplished if charge, Q, and current, I, are measured and analyzed according to Appendix Eqs. (11) to (15). Measurements were made on ten days and the results are shown in Table 3. The measured dwell times and B have coefficients of variation of 0.4%, while $t_{\text{tr}}$ has a 0.1 s (22%) variation. The average B value is 1.005, which means that the measured dwell time is 0.5% greater than the HDR brachytherapy set time. The dose from the transit of the source is equivalent to 0.4 s of dwell time.

**Figure 13:**
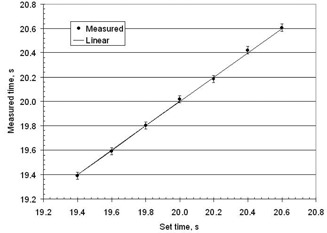
The measured dwell time versus the set dwell time. The points are measured values with error bars that indicate one standard deviation, which are based on seven repeats of the experiment. The solid line is if the measured was equal to the set dwell time.

Setting different dwell times can test the sensitivity of the measurement technique for dwell time. This was accomplished by setting $t_{\text{set}1}$ to 15 s and setting $t_{\text{set}2}$ to different values. The results are shown in Fig. 13. As can be seen, the measured dwell time is equal to the set dwell time within the measurement uncertainty of 0.3 over a 20 s time frame.

**Table 3:** Dwell times measured with the method described by Appendix Eqs. (11) to (15). For these measurements $t_{\text{set}1}$ is 15 s and $t_{\text{set}2}$ is 20 s and $N_{\text{dwell}}$ is 35

*Date of Measurement*	$t_{\text{meas}1}\;(s)$	$t_{\text{meas}2}\;(s)$	*B*	$t_{\text{tr}}\;(s)$
9‐Apr‐13	15.56	20.62	1.011	0.403
11‐Apr‐13	15.51	20.56	1.009	0.373
12‐Apr‐13	15.68	20.68	1.000	0.683
16‐Apr‐13	15.54	20.55	1.002	0.512
18‐Apr‐13	15.50	20.50	1.001	0.475
19‐Apr‐13	15.55	20.57	1.005	0.469
22‐Apr‐13	15.52	20.54	1.003	0.466
23‐Apr‐13	15.47	20.50	1.007	0.358
25‐Apr‐13	15.50	20.51	1.003	0.358
26‐Apr‐13	15.54	20.59	1.008	0.418
Average	15.54	20.56	1.005	0.452
Standard deviation	0.06	0.06	0.004	0.097
Coefficient of variation (%)	0.4	0.3	0.4	21.6

## III. DISCUSSION

About four years ago our clinic decommissioned all film processing equipment, so use of radiographic film for routine HDR brachytherapy quality assurance was no longer feasible. In response to this evolution, new and modified tools have been developed that allow HDR brachytherapy quality assurance tests to be carried out efficiently without film, video cameras, stopwatches, and rulers. The tools and procedures that are described in this work have been in use for daily and quarterly check procedures over the past four years.

This work describes a modification of the insert described earlier.^(9)^ The new insert gives an extended plateau region and a high slope region. Procedures are also described (Spreadsheets in Microsoft Excel format for the various procedures described in this work are available from the author) for making geometric and temporal measurements on a daily schedule and when a source exchange is made. The use of film, video cameras, and stopwatches is avoided. The procedures give results with precision and accuracy of 0.3 mm and 0.3% of the set time.

Since the measurements described in this work are simple to carry out, they can be accomplished with a decrease in the time spent on quality assurance. For the quarterly source exchange quality assurance, the new tool reduces measurement time to about 45 minutes. Previous methods that depended on film would take nearly three hours and did not give the precision that is now directly obtained.

The distance from the first dwell position to the end of the various applicators has been determined for all of the applicators used in our clinic, using the tool, Fig. 1, and the methods described. Data for some typical applicators are shown in Tables 1 and 2. Since the end of the applicators is always easily visible in CT images of patients, dummy seeds are no longer used in our clinic. Since the location of the first dwell position with respect to the end of the applicator has already been determined with the methods in this paper, this location is used in treatment planning.

The distance from the first dwell position to the end of a vaginal‐cylinder straight catheter is adjusted to 5 mm every three months at the time of the source exchange. Other applicators are checked on an annual basis and found to not change within the measurement uncertainty of 0. 3 mm, as shown in Tables 1 and 2. Achieving an accuracy of 0.3 mm in dwell position location takes about 2 min of measuring and analysis time. To this author's knowledge, this level of accuracy is not possible with film analysis and requires at least fivefold more time.

For recommended^4^, ^5^ daily quality assurance tasks, the modifications to the Model 70008, HDR brachytherapy Ir‐192 quality assurance tool has not saved much time, but it does provide for more direct quality assurance measurements with high precision.

Using the equipment described in this work to carry out recommended^3^, ^4^, ^5^ checks of temporal accuracy does not require a stopwatch, but does require an electrometer that has been calibrated in the charge and current modes. The equipment described in this work can be used in an efficient and direct manner to determine linearity and accuracy of dwell times.

The equipment and methods described in this work were for GammaMed HDR brachytherapy equipment in this clinic. Extension of methods and equipment for HDR brachytherapy systems of other manufacturers has not been tested. It would not be difficult to design a template to accommodate applicators for other HDR brachytherapy systems.

## IV. CONCLUSIONS

New and modified equipment and procedures have been developed for measuring HDR brachytherapy dwell position and dwell times efficiently with high accuracy.

The equipment described in this work can be built and modifications can be made in most clinics.
2. Location of dwell position can be determined in straight catheters and ring and ovoid applicators to a precision of 0.3 mm.
3. Timer linearity and accuracy can be determined to a precision of 0.3% over a 20s time frame.
4. Measurement efficiency can be significantly improved over previous methods that used film, video cameras, mechanical rulers, and stopwatches.

## ACKNOWLEDGMENTS

The authors would like to thank Renu Sharma and Jim Reuter for valuable suggestions made during four years of use of this apparatus in clinical measurements. The critical reading and suggestions by Dan Schmidt of Standard Imaging are also greatly appreciated.

## Figures and Tables

**Figure 14:**
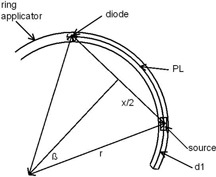
The position template for the ring catheter. The radius of the ring applicator, r, is 16 mm. The distance in the ring of dwell position 1 from the outside surface of the catheter is d1. The distance in the ring, the path length in the ring, from the position of the diode to the outside surface of the tip of the catheter, is set at 10 mm by positioning the catheter against the stop bar shown in Fig. 1.

## Appendix 1: Calculation Formalism

A diagram of the Ir‐192 source and diode geometry is shown in Fig. 2. The distance between the center of the diode and the center of the source is the following:

(App.1) $$d=\sqrt{x^{2}+t^{2}}$$

where *x* is the distance orthogonal to the center of the diode, and *t* is the separation distance between the active area of the diode and the source path in the catheter.

Path length is defined as the distance moved by the source from a known position in the after loader housing. For the GammaMedplus iX HDR brachytherapy system, this known position is the optical path of an optocoupler. Dwell position 1 is the farthest out in the catheter, which is the longest path length. The path length moved by the source when at dwell position number $N_{\text{dwell}}$ can be expressed as follows:

(App.2) $$\text{PL}(N_{\text{dwell}})=\text{PL}1-\text{PLi}\cdot \left(N_{\text{dwell}}-1\right)$$

where *PL1* is the path length moved by the source for dwell position 1, and *PLi* is the path length moved between each dwell position.

For a straight catheter (Fig. 2), x($N_{\text{dwell}}$) for any dwell number $N_{\text{dwell}}$ is the following:

(App.3) $$x\left(N_{\text{dwell}}\right)=10-d1-\text{PLi}\cdot \left(N_{\text{dwell}}-1\right)$$

where *d1* is the distance from the center of the source to the outside edge of the catheter for $N_{\text{dwell}}=1$.

A diagram of the geometry for a ring catheter is shown in Fig. 14. For this geometry, the following holds:

(App.4) $$\beta \left(N_{\text{dwell}}\right)=\frac{\text{PL}}{2\cdot r}=\frac{10-d1-\text{PLi}\cdot \left(N_{\text{dwell}}-1\right)}{2\cdot r}$$

(App.5) $$x\left(N_{\text{dwell}}\right)=2\cdot r\cdot \text{sin}(\beta (N_{\text{dwell}}))$$

where β($N_{\text{dwelll}}$) is the angle shown in Fig. 14 in radians for any dwell number $N_{\text{dwell}},r$ is the radius of the ring catheter, and *PL* is the path length of the source in the ring from the diode. The signal from the diode is expected to change as follows:

(App.6) $$S=\frac{C\cdot \text{exp}(-\alpha \cdot d)}{d^{2}}$$

where *C* is a scaling constant and a is the linear attenuation coefficient for material between the source and the diode. Normalizing S by its maximum value can eliminate the C constant. It is assumed in this modeling of the signal that dose from scatter will be insignificant. The validity of this assumption is demonstrated in the Results section above.

The values of d1 and a are determined parameters. The measured data are fit to Appendix Eqs. (1), (3), and (6) for a straight catheter or Appendix Eqs. (1)‐(6) for a ring catheter, using the solver function in Microsoft Excel, which is a nonlinear regression, steepest descent method.

As previously presented,^(9)^ the current at dwell position x on day D after the source change will be the following:

(App.7) $$I(x,D)=\frac{K(x)\cdot A_{0}\cdot \text{exp}\left(\frac{-0.693\cdot D}{73.8}\right)}{C_{\text{TP}}}$$

where *K(x)* is the function that relates the response of the system to the position x, $A_{0}$ is the source strength at the time of the calibration of the Ir‐192 source, the half life of Ir‐192 decay is 73.8 days,^(13)^ and $C_{\text{TP}}$ is the correction for temperature and atmospheric pressure for the ionization chamber. The decay of source strength on any day compared to the day of the source change can be measured as follows:

(App.8) $$\frac{I(x,D)}{I(x,D=0)}$$

To precisely measure the position of the source one needs a location with a high rate of change in current versus source position. This is the definition of the first derivative of the current versus source position. From Appendix Eq. (7) the following is the first derivative:

(App.9) $$\frac{d(I(x,D))}{\text{dx}}=\frac{d(I(x,D=0))}{\text{dx}}\cdot \text{exp}\left(\frac{-0.693\cdot D}{73.8}\right)$$

Using Appendix Eq. (7), the exponential can be eliminated to obtain:

(App.10) $$\frac{\frac{d(I(x,D))}{\text{dx}}}{I(x^{'},D)}=\frac{\frac{d(I(x,D=0))}{\text{dx}}}{I(x^{'},D=0)}$$

where *x'* is any convenient source position.

Various checks of temporal accuracy can be accomplished if charge, Q, and current, I, are measured since t = Q/I. As previously shown:^(9)^

(App.11) $$t_{\text{meas}}=B\cdot t_{\text{set}}+t_{\text{tr}}$$

where $t_{\text{meas}}$ is the measured dwell time, *B* is a proportionality constant that should be equal to unity if the HDR timer is accurate, $t_{\text{set}}$ is the dwell time set on the treatment console, and $t_{\text{rr}}$ is the transit time that is equivalent to the exposure time as the source travels to the dwell position.

The values of B and $t_{\text{tr}}$ in Appendix Eq. (11) can be determined by using two different $t_{\text{set}},t_{\text{set}1}$ and $t_{\text{set}2}$, as follows:

(App.12) $$t_{\text{meas}1}=\frac{Q(N_{\text{dwell}},t_{\text{set}1})}{I(N_{\text{dwell}})}=B\cdot t_{\text{set}1}+t_{\text{tr}}$$

(App.13) $$t_{\text{meas}2}=\frac{Q(N_{\text{dwell}},t_{\text{set}2})}{I(N_{\text{dwell}})}=B\cdot t_{\text{set}2}+t_{\text{tr}}$$

From Appendix Eqs. (12) and (13) the following is derived:

(App.14) $$B=\frac{\left(t_{\text{meas}2}-t_{\text{meas}1}\right)}{\left(t_{\text{set}2}-t_{\text{set}1}\right)}$$

(App.15) $$t_{\text{tr}}=\frac{t_{\text{meas}1}+t_{\text{meas}2}-B\cdot (t_{\text{set}1}+t_{\text{set}2})}{2}$$
